# The Effectiveness of Cold Atmospheric Plasma (CAP) on Bacterial Reduction in Dental Implants: A Systematic Review

**DOI:** 10.3390/biom13101528

**Published:** 2023-10-16

**Authors:** Ahmed Yaseen Alqutaibi, Abdulbari Aljohani, Abdullah Alduri, Abdulmajid Masoudi, Anas M. Alsaedi, Hesham Mohammed Al-Sharani, Ahmed E. Farghal, Ahmad Abdulkareem Alnazzawi, Afaf Noman Aboalrejal, Abdel-Aleam H. Mohamed, Muhammad Sohail Zafar

**Affiliations:** 1 Department of Substitutive Dental Science, College of Dentistry, Taibah University, Al Madinah 41311, Saudi Arabia; afrgal@taibahu.edu.sa (A.E.F.); anazawi@taibahu.edu.sa (A.A.A.); 2 Prosthodontics Department, College of Dentistry, Ibb University, Ibb 70270, Yemen; 3 College of Dentistry, Taibah University, Al Madinah 41311, Saudi Arabia; TU3702463@Taibahu.edu.sa (A.A.); TU3702857@Taibahu.edu.sa (A.A.); abdulmajidmasoudi@hotmail.com (A.M.); anasmd1416@gmail.com (A.M.A.); 4 National Center for Epidemiology and Population Health, ANU College of Health and Medicine, Canberra 2601, Australia; Hesham.Al-sharani@anu.edu.au; 5 Oral Biology Department, College of Dentistry, Ibb University, Ibb 70270, Yemen; afafaboalrejal@gmail.com; 6 Physics Department, College of Science, Taibah University, Al Madinah 42353, Saudi Arabia; amohamedb@taibahu.edu.sa; 7 Physics Department, Faculty of Science, Beni-Suef University, Beni-Suef 62521, Egypt; 8 Department of Restorative Dentistry, College of Dentistry, Taibah University, Al Madinah 41311, Saudi Arabia; 9 School of Dentistry, University of Jordan, Amman 11942, Jordan

**Keywords:** cold atmospheric plasma, CAP, biofilms, dental implant, bactericidal activity, prosthodontics

## Abstract

Background: The emergence of dental implants has revolutionized the management of tooth loss. However, the placement of clinical implants exposes them to complex oral environment and numerous microscopic entities, such as bacteria. Cold atmospheric plasma (CAP) is often used to treat the surfaces of dental implants, which alters morphological features and effectively reduces bacterial load. Purpose: This systematic review aims to assess the existing literature on the bactericidal properties of CAP when used on various kinds of dental implant surfaces. Review Method: An in-depth examination of MEDLINE/PubMed and EMBASE was performed to identify relevant studies, with the most recent search conducted in May 2023. Studies were selected based on their exploration of CAP’s effects on dental implants compared to control groups, focusing on CAP’s bactericidal efficacy. However, studies that lacked a control group or that failed to measure bactericidal effects were excluded. Results: After applying the selection criteria, 15 studies were ultimately included in the systematic review. The collected data suggest that CAP can effectively reduce bacterial loads on dental implant surfaces, including pathogens like *Streptococcus mitis* and *Staphylococcus aureus*. Furthermore, CAP appears to combat biofilms and plaques that are key contributors to periimplantitis. Conclusion: CAP emerges as a promising treatment option, exhibiting significant bactericidal activity on dental implant surfaces. CAP can decrease the rates of bacterial biofilm and plaque formation, leading to improved outcomes for dental implant patients.

## 1. Introduction

Dental implants have been considered a reliable strategy for tooth replacement for decades, establishing themselves as the gold standard in restoring lost teeth [1]. Unlike other prosthetic treatments, dental implants do not necessitate the preparation of adjacent teeth, therefore maintaining the integrity of natural teeth [2] and improving the quality of life for patients [3]. A wide range of biomaterials has been investigated for the fabrication of dental implants, including titanium, zirconia, and non-degradable polymers such as polyetheretherketone (PEEK) and emerging polyetherketoneketone (PEKK), which are susceptible to bacterial colonization and subsequent infection [4,5].

After implant placement in the patient’s mouth, the dynamic alveolar bone tissues undergo consistent bone remodelling that exposes the threaded implant surface and enhance contamination from the oral environment [6]. Moreover, rapid bacterial colonization and biofilm formation can occur in such scenarios, with the implant surface properties playing a crucial role, leading to peri-implant disease. Peri-implant diseases, including peri-implant mucositis and peri-implantitis, are inflammatory conditions affecting the tissues around dental implants. Peri-implant mucositis is an early stage characterized by soft tissue inflammation, while peri-implantitis involves both tissue inflammation and bone loss [7,8]. Bacterial plaque accumulation on the implant surface is a primary cause of these diseases. Debridement, which involves removing bacterial biofilm and infected tissues, is essential for controlling infection, promoting tissue healing, and preventing disease progression [7]. Decontaminating infected rough implant surfaces may present unique challenges compared to their smooth counterparts. Various decontamination techniques have been investigated for implant surfaces, including laser, chemotherapeutic anti-infective devices, air-abrasive units, and recently cold atmospheric plasma (CAP) [9].

The primary prevention of peri-implantitis diseases and early detection and treatment of inflammation and infection are paramount in patients receiving supportive periodontal therapy following dental implant placement [10]. Treating peri-implant disorders has been the subject of numerous systematic reviews. However, their results are controversial when it comes to the effectiveness of traditional respective and regenerative surgery [7,11,12,13,14,15] as well as preventative treatments [16], nonsurgical treatments [17], adjunctive therapies (antibacterial agents [18], air-polishing [19], and laser [20]). Based on its antibacterial and surface-decontaminating properties, CAP may be an effective tool for treating peri-implantitis. Dental plaque removal on dental implants can present specific anatomical difficulties compared to natural teeth; this highlights the necessity for a therapy with a dependable cleaning efficacy and an efficient penetration depth to kill bacteria in thick biofilm, which can be offered with CAP [21,22].

Plasma is the fourth state of matter and represents an ionized gas that contains ions, electrons, neutral species (atoms and molecules), ultraviolet irradiation, free radicals, and chemically reactive neutral species [23]. Plasma is generally classified into high-temperature thermal and nonthermal subsets [10]. In high-temperature plasma, all particles, including heavy and electron particles, have the same temperature and are in thermal equilibrium. Thermal (quasi-equilibrium) plasma is applicable only in a limited range of temperatures because the gas is heated solely by energy, and its temperatures typically fall within the range of 10,000 to 100,000 K. Finally, nonthermal (non-equilibrium) plasma consists of particles that are not in thermal equilibrium (i.e., cold atmospheric plasma). CAP consists entirely of heavy particles and has a room temperature and an application temperature of roughly 40 degrees Celsius [24]. Air, heliox (a mixture of helium and oxygen), nitrogen, argon, and helium are only some of the gases used to make CAP [25,26].

CAP ions and neutral particles can reach room temperature [27]. Therefore, cold plasmas are used for various biomedical applications, especially in the case of heat-sensitive materials such as biopolymers. In recent years, CAP has been used widely in medicine and dentistry [28].

Several plasma systems have been used to treat contaminated biofilms [29]. These plasma systems can be powered by various energy sources such as direct and alternating current power, microwave and radio frequency power, and pulsed energy sources. In addition, several electrode configurations offer a variety of plasma systems, such as dielectric barrier discharge (DBD), plasma jets, and others. This wide variety of electrode configurations and energy sources has allowed the generated CAPs to address many earlier barriers in plasma applications in biomedical, agricultural, and industrial zones [30].

It is worth mentioning that the use of CAP for dental implant debridement in peri-implant disease is very promising due to its unique benefits. CAP produces a low-temperature ionized gas that kills germs and biofilms without damaging tissues or implants and can penetrate implant surface microstructures and destroy germs to prevent reinfection. In addition, CAP treatment is fast, promotes tissue repair, and reduces inflammation, making it a promising peri-implant disease treatment. Furthermore, CAP is an attractive alternative to other dental implant debridement methods for peri-implant disease due to its non-invasiveness, efficacy against bacteria and biofilms, ability to access difficult areas, and potential tissue healing benefits [9].

There are several published reviews of the antimicrobial effectiveness of CAP [31,32,33]; however, the particular circumstances of CAP’s use in dental implant sterilization call for a comprehensive analysis of the topic because dental implants create a unique biofilm challenge due to their complicated surface geometry and the particular microbial flora of the oral cavity. Moreover, their design makes dental implants prone to bacterial colonization and biofilm formation due to their rough surfaces [34] and micro-gaps [8]. Furthermore, the oral cavity is a distinct ecosystem that hosts a diverse and ever-changing microbial community [35,36]. Because oral bacteria are so diverse and adaptable, the success of CAP sterilization in this setting may vary from its success in others. Finally, enhanced dental implant cleaning has significant clinical consequences because the progression of peri-implant disease due to an infection can necessitate expensive and painful corrective surgery.

Despite the promising outcome of using CAP to manage peri-implant tissues, there is no systematic review reporting the potential antibacterial effects of CAP on dental implants. Therefore, this review will fill a need in the literature by shedding light on antibacterial effect of CAP on dental implant surfaces.

## 2. Review Method

This systematic review methodology adhered to the guidelines outlined by the PRISMA statement [37]. We defined a targeted research question to guide our investigation: “Does the in vitro application of cold atmospheric plasma show an antimicrobial effect on microorganisms compared to no treatment when applied to the dental implant surface?”

This systematic review was registered in the Open Science Framework (OSF) registries (https://doi.org/10.17605/OSF.IO/JN8WS).

The results were restricted to research articles written in English. The keywords for the search were: (“cold atmospheric plasma” OR “nonthermal plasma” “nonthermal atmospheric plasma” OR “cold atmospheric pressure plasma” OR “argon plasma” OR “helium plasma” OR “oxygen plasma” OR “nitrogen plasma” OR “air plasma” OR “plasma gases” OR “plasma jet” OR “dielectric barrier discharge” OR “glow discharge” [MeSH Terms]) AND (“dental implant” OR “oral implant” OR “oral implantology” OR “Osseointegrated implants”) AND (“disinfection” OR “sterilization” OR “bacterial inactivation” OR “bactericidal” OR “bacteriostatic” OR “microbicidal” OR “antimicrobial”).

The electronic databases MEDLINE/PubMed, Cochrane, and EMBASE were independently screened by two reviewers (M.A.A and A.Y.A). There were no language restrictions, and the search covered studies up to May 2023.

Studies were considered for inclusion if reported findings on the antimicrobial properties of cold plasma on dental implants, compared to no or other treatments. However, studies lacking a control group or failing to measure antimicrobial effects were excluded from the review.

Search results from the electronic databases were consolidated using the Endnote X 20.4.1 reference manager software (Clarivate Analytics, PA, US). Prior to the screening, any duplicated records were eliminated. The titles and abstracts of the selected studies were independently screened by the two reviewers (M.A.A. and A.Y.A.). Studies meeting the criteria were subjected to a full-text review for final inclusion. Further potential sources were sought by manually screening shortlisted studies’ reference lists and additional relevant Web of Science articles.

Assessing the risk and examining the quality of each publication was accomplished using a specially crafted assessment instrument hinging on US EPA guidelines governing the bactericidal activity of disinfectants [38,39,40,41]. This tool comprises eleven items (Table 1), with nine items scoring one point each and two items with a value of two points each, making the maximum possible score thirteen points. A percentage was used to represent the final score, which was calculated by dividing the points earned by the total possible points. This tool assessed the essential stages of the study protocol and its repeatability to account for the potential overestimation of outcomes.

## 3. Results

**Figure 1:**
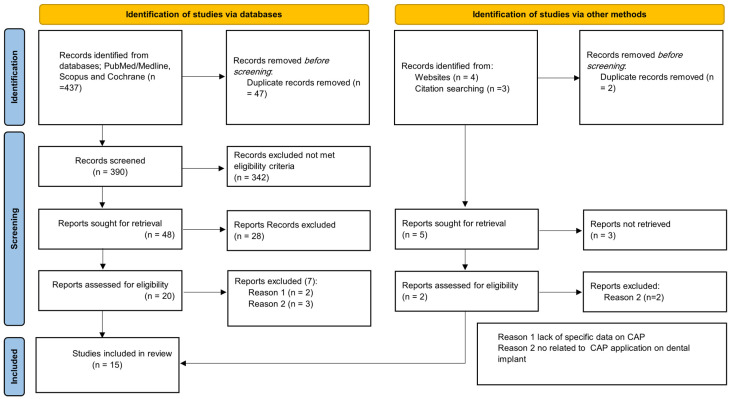
Flow diagram illustrating the study selection phases based on Preferred Reporting Items for Systematic Reviews and Meta-Analyses guidelines (PRISMA).

A total of 15 studies were included; the selection process is presented in Figure 1. Table 2 includes the detailed characteristics of the reviewed studies.

CAPs, particularly those employing argon, have demonstrated significant results in sterilizing and treating biofilms formed by *Streptococcus mitis*, the bacteria commonly found in infected peri-implants. The results of Canullo et al. [42] demonstrated that plasma successfully disinfected the implant surfaces with complete microbiota elimination observed after 120 s of treatment. Even after a shorter exposure of 30 s, a significant reduction in bacterial load was observed. Conversely, untreated control samples exhibited growth of a higher bacterial count on their surfaces.

Similarly, Preissner et al. [43] utilized a tissue-tolerable CAP to examine its bacterial efficiency [17]. They reported a lower colony forming unit (CFU) count in implants treated with this plasma than their control counterparts. Additionally, no fluorescence, often indicative of bacterial presence, was detected on the sterile implant surfaces [17].

Further research has focused on the impact of CAP treatment on *Escherichia coli* (*E. coli*) biofilms. Researchers exposed *E. coli* biofilms to CAP and then evaluated the biofilm eradication on implant material surfaces using an XTT and a safranin assay [44]. The results demonstrated effective inactivation of 1-day-old *E. coli* biofilms on various substrates. Remarkably, plasma treatment for 3 min inactivated approximately 95% of *E. coli* biofilms across all biomaterials tested, and there were no significant variations in the bacteria inactivation rate for treatments ranging from 1 to 3 min [18]. Moreover, the dielectric barrier discharge plasma treatment significantly reduced the extracellular polymeric substance (EPS) of *E. coli* biofilms developed on titanium discs that almost completely inactivated the bacteria [18]. The plasma therapy managed to inactivate almost all *E. coli* present in biofilms within three minutes and prevented up to 50% of biofilm formation after a day [18].

Two studies [44,45] conducted experiments on *Staphylococcus aureus* (*S. aureus*) bacteria using CAP devices from Advanced Plasma Solutions, Malvern, PA, USA, and Plasma Medical Systems, Bad Ems, Germany, applying treatments to 76 titanium discs [18,19]. One of these studies reported a significant effect of plasma treatment: 95% of *S. aureus* present in biofilms were inactivated after a mere 3 min of plasma treatment. Moreover, the study found that CAP could prevent up to 50% of biofilm formation one day post-treatment and disrupt the extracellular polymeric substances secreted by the bacteria [18]. Similarly, another study showed that CAP successfully inactivated more than six logs of *S. aureus* biofilm formed on sandblasted, large grit, acid-etched (SLA) titanium surfaces after 7 days, proving to be superior to both noncontact and contact laser treatments in terms of antibiofilm efficacy [44].

The impact of plasma treatment on *Porphyromonas gingivalis* (*P. gingivalis*) bacteria was examined in two additional studies [8,46]. These studies applied helium gas air plasma to the bacteria and evaluated the effect of cold atmospheric plasma on implant surface decontamination. The titanium discs measuring 10 mm in diameter and 1 mm in thickness were used in a study by Lee et al. (2019) [8], where *P. gingivalis* was raised for six days in an anaerobic environment [46]. Atmospheric-pressure plasma jets (APPJs) were applied to the acid-etched titanium and sandblasted discs using helium gas. The amount of *P. gingivalis* significantly decreased as a result. After 3 and 5 min of treatment, no intact bacteria were found, suggesting that the treatment had not only cleansed the SLA surface but had also damaged the bacterial biofilm structure. In contrast, the control group showed persistent bacterial biofilm after 5 min of treatment.

In a study by Kamionka in 2022 [46], CAP was employed to cleanse plaque from 280 titanium disc samples characterized by robust biofilms sourced from a volunteer suffering from periodontal disease with deep pocket subgingival plaque. The biofilms were grown for seven days at 37 °C using Dulbecco’s modified Eagle’s medium (DMEM), which was changed every 24 h. A plasma jet (kINPen) was employed on the titanium discs using argon gas at 2–6 kV and a 5 slm flow rate, 3.5 W power of plasma, and a 1 kHz frequency for 9 min. The biofilm was evaluated post-treatment (Day 0) and immediately following 5 days of incubation. On day 0, only a slight decrease in biofilm fluorescence levels was seen on both surfaces compared to the surfaces where biofilm control was present. However, on day 5, the reassessment of the biofilms indicated that CAP treatment was highly influential in biofilm removal compared to other methods. Furthermore, a combination of CAP and air polishing showed promising results.

Another study by Yang et al. (2019) [47] used a helium CAP with 2.85 kV, 17 kHz, and a 13.5 slm flow rate on yttrium-stabilized zirconia discs. After incubating *P. gingivalis* under standard anaerobic conditions, the researchers observed a decrease in bacterial counts after 24 h cultivation among the untreated group and those treated for 30, 60, and 90 s. The same trend was observed for 48 h and 72 h cultivation periods, suggesting a long-lasting inhibition effect [8]. The number of bacteria that adhered to the materials’ surface was decreased with longer treatment times, indicating a significant reduction in bacterial colonization [21].

The impact of plasma treatment on *Streptococcus mutans* (*S. mutans*) bacteria was examined by Yang et al. [21], *S. mutans* were subjected to helium cold atmospheric plasma (CAP) treatment with a flow rate of 2.85 kV, 17 kHz, and 13.5 slm on yttrium-stabilized zirconia discs. The bacteria were cultivated under standard conditions (5% CO_2_, 95% humidified air, at 37 °C). The researchers investigated the bacteria’s response, including adhesion, morphology, biofilm formation, and survival. A substantial difference was observed after 24 h of cultivation between the untreated and treated groups. There was a marked reduction in biofilm formation in the 30, 60, and 90 s treatment groups. This trend continued after 48 and 72 h of cultivation, showing that biofilm formation decreased as the plasma treatment time increased [21].

Meanwhile, Flörke et al. [48] compared the efficiency of three adjunctive therapy alternatives (CAP, photodynamic therapy (PDT), and 35% phosphoric acid gel (PAG)) in removing contaminants from titanium implant surfaces. Their findings revealed that the CAP implants had the fewest bacteria, and CFU is higher in implants with PDT treatment. Positive control groups showed less CFU on plasma-treated implants. The PDT and PAG groups did not differ significantly from one another compared to the PDT and CAP groups.

None of the three treatment approaches eradicates microorganisms from the implant surface. Comparably, image sections in all three groups had no bacteria. However, the PDT and PAG groups did not show any discernible differences. Additionally, the titanium surface was unaffected by any of the three therapy strategies [22].

Idlibi et al. 2013 and Rupf 2011 [9,49] formed oral plaque biofilms in situ on titanium discs over 24–72 h. Both studies used 2.45 GHz nonthermal microwave-driven CAP, plasma jet mode, and helium gas flow with a mean power of plasma jet (3–5 W) were performed with a thermal resolution of 0.1 °C at room temperature, a frame rate of 100 Hz, and an optical resolution of 160 *×* 120 pixels. According to Idlibi et al. [24], the percentage cover of green fluorescence was 90% and 78% for untreated and gas control, respectively. A significant (*p* = 0.008) decline in the green fluorescence percentage cover between 38% and 15% was seen after treatment with CAP. Rupf, 2011, [49] demonstrated that both 24 h and 72 h biofilms had significant reductions in biofilm levels and vitality (green fluorescence) after the application of CAP. The study’s results demonstrated that biofilm destruction on titanium in situ can be significantly reduced using CAP. Comparatively, exposure to CAP decreases the viability and amount of biofilm compared to a positive control treatment [24,49].

In two studies [36,50] published by Lai Hui et al. in 2020 and 2021, plasma treatment was carried out using a spark plasma pen with a frequency of 1.4 Hz and a voltage of 10 kV on titanium implant and disc. The first investigation combined erythritol-based air abrasion (AA) with cold atmospheric plasma. In the second study, air abrasion using erythritol powder in a liquid medium was combined with cold atmospheric plasma. The outcomes demonstrated that AA and CAP are very efficient at removing biofilm from various titanium surfaces. The structure of the titanium surface remained unaltered, and no specific effects of CAP were seen.

A study performed by Matthes et al. [51] used different CAP modalities to remove 7-day-old biofilm on titanium discs to evaluate cell spreading after 5-day treatment. The biofilms were grown and harvested from subgingival plaque from a deep pocket of a periodontally diseased volunteer. The plaque suspension was cultivated in an incubator for seven days at 37 °C for 24 h, while the medium was replaced every 24 h. The study used plasma jet (kINPen) argon gas with 2–6 kV, 5 Slm, 3.5 W plasma power, and 1 kHz on acid-etched, sandblasted, and sterile titanium discs. The 5 days of cultivation results in biofilm and cell growth regrowth. Cell coverage on AP(air polishing) + CAP(300 s) (75.2 18.1%), AP + CAP(720 s) (57.5 18.7%), positive control (77.7 21.2%), negative control, and both CAP groups were significantly different from one another. No cells were to be found on biofilm and CAP control discs. The difference in the bacterial load was borderline significant (*p* = 0.046).

In a study by Duske et al. [52], CAP was utilized to eliminate plaque cultivation on sandblasted-etched titanium discs. These discs were placed in 96-well microtiter plates containing 100 mL of subgingival plaque derived from a deep pocket in a periodontally diseased volunteer. The discs were incubated for seven days at 37 °C, changing the media every 24 h. After incubation, 0.9% sodium solution was used to rinse the discs after discarding the medium. For the treatment, a plasma jet (kINPen) was utilized, harnessing argon gas with a range of 2–6 kV and a 5 slm flow rate, operating at 2–3 W plasma power and a frequency of 1.82 kHz. This study found that CAP was effective in decontaminating implants and also noted an improvement in osteoblast growth on titanium discs covered by biofilm [27].

**Table 2:** Characteristics of the reviewed studies [9,29,36,42,43,44,45,46,47,48,49,50,51,52,53].

Study ID	Year	Device	Plasma Mode	Gas	Device Parameters				Implant Material	Sample Size	Control	Incubation (hours)	Species
					Power (W)	Voltage (kV)	Frequency (kHz)	Gas Flow Rates (slm)					
Canullo et al.	2017	argon atmospheric pressure dielectric barrier discharge	Plasma Jet	Argon	8	NR	NR	None	Titanium Grade 4 discs (Sweden and Martina)	720 discs	Untreated titanium discs	24	*Streptococcus mitis*
Duske et al.	2015	(kINPen08, INP Greifswald, Germany)	Plasma Jet	Argon (99%) with 1% oxygen	2–3	2–6	1.82	5	Titanium Grade 4, diameter 15 mm, and thickness 1 mm Straumann, Freiburg, Germany)	80 discs	Discs without biofilm, untreated biofilm and autoclaved biofilm	24 & 120	Sub-gingival plaque from deep pockets
Flörke et al.	2022	kINPen^®^ MED (neoplas tools GmbH, Greifswald, Germany)	Plasma Jet	NR	5	NR	NR	NR	Titanium (TiPure Plus BEGO Semados^®^ SC, BEGO GmbH & Co. KG, Bremen, Germany, 3.75 × 8.5 mm)	45 implants	Negative: 2 implants neither infected nor decontaminated + 2 implants had been kept. free of contamination and treatment. Positive: before the decontamination procedure, one implant was removed.	24	*Enterococcus faecalis*
Ibis et al.	2016	Advanced plasma Solutions, Malvern, PA, USA	Atmospheric Pressure	NR	0.29	31	1.5	None	Steel, titanium, and polyethylene rods cut into disc	NR	Untreated disc as positive control	24	*Escherichia coli* and *Staphylococcus aureus*
Jungbauer et al.	2022	piezobrush^®^ PZ3; Relyon Plasma, Regensburg, Germany	Plasma brush	NR	8	Non	50	NR	Polystyrene, dentin, titanium	60	NR	84	12 bacterial strains
Kamionka et al.	2022	kINPen^®^	Plasma Jet	Argon	3.5	2–6	1	5	Titanium discs (Nobel Biocare AB, Göteborg, Sweden’s TiUnite) 5 mm in diameter and 1 mm in thickness	280 discs	Untreated discs with biofilm and sterile discs	168	Subgingival plaque
Lai Hui et al.	2021	Atmospheric experimental plasma pen jet	Plasma Jet	NR	NR	10	1.4	NR	Grade 4 titanium discs diameter 10 mm, thickness 1.5/2 mm with two different surfaces	112 discs	Negative control: treated titanium discs that have not been contaminated. Positive control: contaminated and untreated discs	96	10% of a patient’s peri-implantitis human saliva
Hui et al.	2021	NR	Plasma Jet	NR	NR	10	1.4	NR	Dental implants made of grade 4 pure Ti	35 implants	Negative (2 S non-contaminated, treated by AA and CAP) Positive control group: (3 S untreated, contaminated)	96	Saliva from peri-implantitis patient
Lee et al.	2019	Dawonsys, Ansan, Republic of Korea’s MF plasma power supply	Plasma Jet	Helium (He)	NR	7	10	5	Grade 4 titanium discs (Osstem Implant Co., Ltd., Busan, Republic of Korea) 10 mm diameter, 1 mm thickness	12 discs	Untreated discs	144	*Porphyromonas gingivalis*
Matthes et al.	2017	neoplas GmbH, Greifswald, Germany, kINPen 09	Plasma Jet	Argon	3.5	2–6	1		Grade 4 titanium discs (BIOMET 3i LLC, Palm Beach Garden, FL, USA)	18 discs	Negative control: untreated biofilm Positive control: sterile pristine discs	24	MG63 cells, bacteria, or biofilm are present
Ulu et al.	2018	Bad Ems, Germany, Plasma Medical Systems	NR	NR	5	7	1.2	None	Large-grit, acid-etched (SLA), sandblasted titanium discs	76 discs	Er: YAG laser	168	*Staphylococcus aureus*
Idlibi et al.	2013	Leibniz Institute of Surface Modification, Leipzig, Germany	Plasma Jet	Helium, O2	3–5	None	2.45	2	Titanium discs	200 discs	untreated and treated controls (diode laser, air-abrasion, chlorhexidine)	72	Oral biofilms
Preissner et al.	2016	Tissue tolerable plasma (TTP120)	Plasma jet	Argon gas	2	10–15	28: direct sonication 35: indirect sonication	4.3	Titanium (2.5 × 13 mm tiny Implant, Biotechnology Institute BTI, Miñano, Spain, REF: IRT2513)	32 implants	Negative control: rinsed with 1 NaCl, Positive control: irradiated with diode or plasma	84	*Strepto-coccus Mitis*
Rupf et al.	2011	custom-built (Leipzig, Germany, Leibniz Institute of Surface Modification)	plasma jet	Helium	3–5	None	2.45	None	Titanium discs grade 2, friadent, 5 mm in diameter and 1 mm in thickness, Mannheim, Germany	Total 334 298 with biofilm 36 without	Biofilms with and without water/air treatment.	72 and 24	Oral cavities biofilm
Yang et al.	2020	Cold atomosphirc plasma	Plasma jet	Helium	0.95	2.85	17	13.5	Yttrium-stabilized zirconia discs (Wieland, Pforzheim, Germany)	24 discs	The control group left untreated.	72	*Streptococcus mutans*, *Porphyromonas gingivalis*

The quality assessment had values ranging from 30% to 92% (Table 3). We rated two articles at less than 50%. There was an excellent inter-rater agreement (=0.91). A meta-analysis was not feasible because of the wide variation in outcomes caused by differences in device characteristics and experimental conditions.

**Table 3:** Assessment of the included studies. The score was calculated by summing each point (score = sum/13 ∗ 100) [9,29,36,42,43,44,45,46,47,48,49,50,51,52,53].

Study ID	Item 1	Item 2	Item 3	Item 4	Item 5	Item 6	Item 7	Item 8	Item 9	Item 10	Item 11	Total	Score
Canullo et al.	1	2	1	1	1	0	1	1	1	1	1	11	84.6
Duske et al.	1	1	2	0	1	1	0	1	1	1	0	9	69.2
Flörke et al.	1	2	1	1	0	1	1	1	1	1	0	10	76.9
Ibis et al.	0	2	0	1	0	0	1	0	0	0	0	4	30.8
Jungbauer et al.	1	1	2	1	0	0	1	1	0	1	0	8	61.5
Kamionka et al.	1	2	2	1	0	1	1	1	1	1	1	12	92.3
Lai Hui et al.	1	1	1	1	1	0	1	1	1	1	0	9	69.2
Lai Hui et al.	1	1	1	1	0	1	1	1	1	1	1	10	76.9
Lee. et al.	1	2	0	0	0	0	1	1	1	1	0	7	53.8
Matthes et al.	1	2	1	1	0	1	1	0	1	0	1	9	69.2
Ulu et al.	1	1	1	1	0	0	1	1	1	1	0	8	61.5
Idlibi et al.	1	2	1	1	0	0	1	1	1	1	0	9	69.2
Preissner et al.	0	1	1	0	1	0	1	1	1	1	1	8	61.5
Rupf et al.	0	1	1	0	0	1	1	0	0	1	0	5	38.5
Yang et al.	0	1	1	0	1	1	1	1	0	1	1	8	61.5

## 4. Discussion

The present systematic review demonstrates that CAP significantly reduces bacterial colonization on the dental implant surface, as inferred by included studies investigating CAP’s effectiveness on bacterial reduction in dental implants using various plasma sources, treatment times, and bacterial strains.

The use of cold atmospheric plasma (CAP) in dentistry, particularly for dental implants, has recently sparked considerable attention. Numerous in vitro experiments [29,42,43,44,45,46,48,50,53] have investigated CAP’s bactericidal activity as a novel decontamination method for dental implant surfaces.

Dental implants are prone to bacterial colonization, leading to peri-implant diseases such as peri-implantitis, which can result in implant failure [7,54]. CAP’s bactericidal property has been demonstrated against a wide range of microorganisms, such as *Streptococcus mutans* [47], *Porphyromonas gingivalis* [29], and *Streptococcus mitis* [43], which are often implicated in dental implant infections. In addition to its bactericidal effect, CAP has been shown to improve the surface characteristics of dental implants, enhancing osseointegration via boosted surface wettability and promoting osteoblasts’ adhesion and protein adsorption [42,52]. This dual benefit makes CAP a highly promising dental implantology tool, potentially reducing infection rates and implant failures. Moreover, since the temperature of atmospheric cold plasmas stays relatively close to room temperature, it could be used to sterilize bacteria and biofilms without damaging tissues or implants. It can penetrate relatively thick biofilms and access challenging locations [9].

The reactive components of CAP, including reactive oxygen and nitrogen species (RONS), charged particles, and UV radiation, can compromise the integrity of bacterial cell walls, coats, and membranes. This leads to the inactivation of microbes and the disruption of biofilm structures [55].

The removal of bacterial biofilms is the primary goal of peri-implantitis treatment. However, removing periodontal biofilms becomes challenging because of the implant’s design and rough surface texture [12]. Sandblasted, large grit, acid-etched implant surfaces (SLA) are more complex than machined surfaces to decontaminate [29]. CAP has been compared to other decontamination methods commonly used in dentistry, such as lasers, air abrasion, and chlorhexidine. The results have shown that CAP can achieve comparable or even superior biofilm removal compared to these modalities. This highlights the potential of CAP as an effective alternative or adjunctive treatment for biofilm-related complications [9]. On the other hand, Dusk et al. [52] combined mechanical treatments with CAP that demonstrated synergistic antimicrobial effects. The mechanical disruption of biofilms and the antimicrobial properties of CAP enhance the overall effectiveness of biofilm removal and decontamination. This combination approach may be particularly beneficial in cases of peri-implantitis, where biofilm removal is crucial for successful treatment outcomes.

Cold plasma has emerged as a promising modality in treating preimplant diseases due to its remarkable efficacy, particularly its antibacterial effect. Cold plasma offers several advantages over other modalities, such as lasers, chemicals, and mechanical debridement. Firstly, cold plasma has a broad-spectrum antimicrobial action, effectively targeting many bacteria and even antibiotic-resistant strains. This makes it an invaluable tool in combating infections associated with preimplant diseases. Cold plasma treatment is also nonthermal, ensuring no or minimal damage to surrounding healthy tissues while achieving effective microbial control. Unlike chemicals, which may have limitations due to toxicity or potential side effects, cold plasma is a safe and non-toxic option.

Furthermore, cold plasma can reach difficult-to-access areas and penetrate biofilms notorious for resisting conventional treatments. This makes it particularly useful in eliminating persistent bacterial colonies in pre-implant diseases. Overall, the outstanding antibacterial efficacy of cold plasma, coupled with its safety and ability to target hard-to-reach areas, positions it as a highly effective modality in treating preimplant diseases.

There are limited studies that evaluate the CAP application in vivo. The adjunctive use of CAP significantly increased peri-implant bone levels and reduced inflammation three months after therapy in a model of peri-implant disease induced through ligament injuries in beagle dogs, compared to conventional treatment with plastic curettes. This group also had significantly lower Tannerella forsythia and *P. gingivalis* levels than the control group [56]. Substantially more significant attachment level gain in severe periodontal pockets and a reduced load of periopathogens were seen in individuals treated with adjunctive CAP compared to standard treatment three months after therapy [57].

Despite its hopeful implications, CAP’s clinical significance in dental implantology is impeded by several constraints. Most importantly, the vast majority of investigations conducted to date have been in vitro, restricting their translation into clinical practice. In vitro studies frequently fail to recreate the complex environment of the mouth cavity, where factors such as saliva, blood, host immune response, and microbial interactions can significantly impact bacterial survival and antimicrobial efficacy [58]. For example, in the presence of organic material, which is prevalent in the oral environment, the bactericidal effect of CAP can be diminished.

Furthermore, the long-term safety of using CAP on dental implants and adjacent tissues is mainly unknown. Although preliminary investigations show low harmful effects, long-term in vivo research is required to validate this. Likewise, there is a lack of established protocols for CAP application, which includes differences in treatment time, plasma-generating technologies, and distance from the target surface [31]. This diversity makes it difficult to compare outcomes across research and generate evidence-based clinical guidelines.

To address these shortcomings, well-designed in vivo studies and clinical trials testing the safety and efficacy of CAP in dental implantology are urgently needed. Such studies should take into account the complicated oral environment and include standardized CAP administration techniques. Furthermore, research should concentrate on determining the precise mechanisms of CAP’s bactericidal activity. This insight will aid in the optimization of CAP utilization and may reveal new applications in dentistry.

Overall, this systematic review highlights the promising antibacterial effects of CAP on dental implant surfaces, emphasizing its potential for improving the long-term success of dental implant therapy. CAP represents an innovative approach to combatting peri-implant infections and holds promise as a valuable adjunctive treatment option in clinical implant dentistry.

## 5. Conclusions

According to the existing evidence from in vitro studies, CAP has shown promise as an effective biofilm removal and decontamination method in dental implantology. Its ability to disrupt biofilm structures, its comparative efficacy to other decontamination modalities, and its potential for synergistic antimicrobial effects make it a valuable tool in combating biofilm-related complications. Further studies and advancements in CAP technology are expected to refine treatment protocols and expand its applications in implant dentistry. Variability in outcome-deciding elements such as specimen preparation, inoculum size, biofilm growth time, and treatment periods must be reduced by standardizing the experimental setup to compare the activity of different devices. The antibacterial effects of biofilms composed of only one bacterium species have been the primary focus of research. More challenging and clinically significant are multi-species biofilms. There needs to be further study using intentional biofilm compositions. Last but not least, these in vitro results need to be verified in animal and clinical studies.

## Figures and Tables

**Table 1:** Criteria for the assessment of the included studies.

Items	Points Attributed According to the Response for Each Critical Step
1. Preparation of microorganisms	1 if described 0 if not described
2. Technical data of plasma generator	2 if at least 3 parameters described or commercially device 1 if at least 1 parameter described 0 if not described
3. Experimental size presented	2 for theoretical + true inoculum sizes 1 for theoretical inoculum size 0 if not described
4. Experimental temperature	1 if described 0 if not described or over 47 °C
5. Protection of samples	1 if described 0 if not described
6. Micro-organisms recovery	1 if other method with mechanic action and validated with a test 0 if not clearly described or technic not validated
7. Time, temperature and method indicated	1 if described 0 if not or poorly described
8. Culture media	1 if described 0 if not described
9. Number of experiments	1 if described with more than one experiment 0 if not described or described with onlyone experiment
10. Statistical method (to compare differences)	1 if described 0 if not described
11. Declaration	1 if declared 0 if not declared

## Data Availability

Data are available upon request from authors.
